# The weekend effect: does time of admission impact management and outcomes of small bowel obstruction?

**DOI:** 10.1093/gastro/gou043

**Published:** 2014-07-09

**Authors:** Derek P. McVay, Avery S. Walker, Daniel W. Nelson, Christopher R. Porta, Marlin W. Causey, Tommy A. Brown

**Affiliations:** Department of Surgery, Madigan Healthcare System, Tacoma, WA, USA

**Keywords:** small bowel obstruction, weekend effect, time of admission, outcomes

## Abstract

**Aims:** To determine whether day and time of admission influences the practice patterns of the admitting general surgeon and subsequent outcomes for patients diagnosed with small bowel obstruction.

**Methods:** A retrospective database review was carried out, covering patients admitted with the presumed diagnosis of partial small bowel obstruction from 2004–2011.

**Results:** A total of 404 patients met the inclusion criteria. One hundred and thirty-nine were admitted during the day, 93 at night and 172 on the weekend. Overall 30.2% of the patients were managed operatively with no significant difference between the groups (*P* = 0.89); however, of patients taken to the operating room, patients admitted during the day received operative intervention over 24 hours earlier than those admitted at a weekend, 0.79 days *vs* 1.90 days, respectively (*P* = 0.05). Overall mortality was low at 1.7%, with no difference noted between the groups (*P* = 0.35). Likewise there was no difference in morbidity rates between the three groups (*P* = 0.90).

**Conclusions:** Despite a faster time to operative intervention in those patients admitted during the day, our study revealed that time of admission does not appear to correlate to patient outcome or mortality.

## INTRODUCTION

Small bowel obstructions are a common part of general surgery practice. Management of bowel obstructions typically involves a period of non-operative management, which often varies based upon the timing of their presentation and admission. This is important, as small bowel obstructions (SBO) represent a common reason for emergency room visits, hospitalizations, and patient morbidity and mortality, comprising nearly 15% of all emergency room visits for abdominal pain and roughly 300 000 hospital admissions yearly in the United States [1, 2]. Recently, there have been multiple studies analysing the “weekend effect”: that is, do patient management and outcomes differ, based on the time of admission? The common assumption is that decreased staffing and cross-coverage on weekends and nights probably results in delayed operative intervention and increased morbidity and mortality; however, thus far there has not been a unified answer as to whether patients admitted on a weekend or at night fair worse than those admitted during the day.

Over the last decade, multiple studies have analysed this effect and found conflicting data, with some reporting worse outcomes in those admitted during the night/weekend period while others have revealed no difference [3–8]. Crowley and colleagues reported a 12% increased mortality for patients admitted on a weekend with an intra-cerebral hemorrhage, while others, such as Khanna and colleagues, showed no difference in medical patients’ outcomes for weekend admissions [9, 10]. The majority of studies reporting worse outcomes in patients admitted during the weekend have been concerned with patients who required urgent or emergency intervention; for instance, Worni and colleagues reported worse outcomes (increased re-operation and post-operative complications) in patients undergoing surgery for diverticulitis who were admitted on a weekend [8].

These studies, when looking at the “weekend effect,” have analysed many time-sensitive diseases and pathologies including gastrointestinal hemorrhage, stroke, and mortality in critical care admission [3–6, 9]; however, to date there has not been an analysis of the effect that time of admission has on patients admitted with SBO. The management of SBO patients has proven to be time-sensitive and delay in diagnosis and/or treatment can have significant untoward effects [11, 12]. The aim of this study was to evaluate whether night and/or weekend admissions for small bowel obstructions make any difference in time to operation and indicate whether these patients are at increased risk for adverse outcomes; specifically analysing 30-day mortality and inpatient morbidity.

## METHODS

After approval by our institutional review board, we conducted a retrospective database review of all adult patients who presented to our emergency room between 2004 and 2011 and were admitted with a presumed diagnosis of a small bowel obstruction (complete and partial). We then examined each electronic medical record and extracted basic demographic data, date and time of admission, essential emergency room clinical data (vital signs, physical exam signs, emergency room interventions), total hospital days, time to operation (for those patients managed operatively), 30-day mortality and specific in-hospital complications. Post-operative complications included repeat operation, transfer to the intensive care unit (ICU), hemorrhage, renal failure, stroke, acute myocardial infarction, pneumonia and sepsis.

Patients were then categorized into three groups, based on time of admission. Those admitted between the hours of 07.00 and 19.00 Monday through Friday were classified as day-time admissions. Those admitted between the hours of 19.01 and 06.59 Monday through Thursday were classified as night-time admissions and those admitted between 19.01 Friday night and 06.59 Monday morning were considered weekend admissions. Missing data for demographic variables of interest were checked for any significant variance from the study population and then excluded for evaluation of that data element only. Categorical variables were represented as a rate with statistical analysis by chi-squared test and continuous variables as mean +/− standard deviation and analysed by student’s *t*-test. Variables which reached statistical significance in the univariate model (*P* < 0.05) and other demographics *a priori* (based on prior studies and expert opinion) were then entered into a logistic regression model to identify independent factors associated with an increased risk of complications, mortality, and operative intervention. All data analysis was performed using PASW 19.0 software (SPSS Inc., Chicago, IL) with the results presented as adjusted odds ratios (OR) with 95% confidence intervals (95% CI) and statistical significance for this study was set at an alpha of 0.05.

## RESULTS

During the specified time period, 404 patients met the specific inclusion criteria. Day admissions totaled 139, while night and weekend totals were 93 and 172, respectively. The majority of the population were female at 58% and the mean age was 62 ± 17 (range 18–95).

The average length of stay (LOS) for all patients admitted was 5.77 ± 7.49 days (range 1–108). Comparing timing of admission, day admissions remained in hospital 4.9 ± 4.5 days; night admissions stayed 6.2 ± 5.5 days, and weekend admissions stayed 6.2 ± 9.9 days (*P* = 0.27) (Table 1). However, when stratified by day *vs* night and weekend, there was a difference in hospital LOS (4.9 ± 4.5 *vs* 6.2 ± 8.6 days respectively; *P* = 0.05). With operative management, the average LOS was 10.1 ± 11.5 days and 3.9 ± 3.5 days for those managed non-operatively (*P* < 0.001).

**Table 1.** Management and outcomes

	All patients	Day	Night	Weekend	*P*-value
	(*n = *404)	(*n = *139)	(*n = *93)	(*n = *172)	
Length of stay (days)	5.77 ± 7.49	4.9 ± 4.5	6.2 ± 5.5	6.2 ± 9.9	0.27
Operative management (*n*, %)	122 (30.2%)	41 (29.5%)	30 (32.3%)	51 (29.7%)	0.89
Time to operation (days)	1.38 ± 2.30	0.79 ± 1.37	1.37 ± 2.31	1.9 ± 2.78	0.06
Need for bowel resection (*n*, %)	122 (30.2%)	41 (29.5%)	30 (32.3%)	51 (29.7%)	0.78
Overall complication rate (*n*, %)	41 (10.1%)	13 (9.4%)	10 (10.8%)	18 (10.5%)	0.90
Operative complication rate (*n*, %)	38 (31.0%)	13 (31.7%)	8 (26.7%)	17 (33.3%)	0.82
Mortality (*n*, %)	7 (1.7%)	2 (1.4%)	2 (2.2%)	3 (1.7%)	0.35

Patients were managed operatively 30% of the time and the mean time to operation was 1.38 days. There was no significant difference in admission timing based on whether or not a patient needed an operation; 29.5, 32.3, and 29.7% (day/night/weekend, respectively; *P* = 0.89) (Table 1). However, patients admitted during the day were taken to the operating room a full day sooner than those admitted on a weekend (0.79 days *vs* 1.90 days; *P* = 0.05). Thirty-seven patients underwent bowel resection with no significant difference between the three groups (10.1, 8.6, & 8.7% day/night/weekend, respectively; *P* = 0.78) (Table 1).

Multivariate analysis revealed that patients with an elevated lactate (greater the 2.2 mmol/L) were 5.6 times more likely to undergo a bowel resection when compared to those with normal lactate levels (OR = 5.6 [1.5–20.6]; *P* = 0.009). Trending towards significance, tachycardia (heart rate >100 beats per minute) tripled one's risk for bowel resection (OR = 3.3 [0.99–11.1]; *P* = 0.051). When analysing whether time to surgery affected outcomes there was no statistical significance with regard to any complication or mortality (*P* = 0.43 & 0.24, respectively). The only clinical finding associated with a need for operation was peritoneal signs in the emergency room (*P* ≤ 0.05). Interestingly, computed tomography (CT) scan on admission and placement of a nasogastric tube inserted in the emergency room almost tripled and doubled the risk of operative intervention respectively (*P* = 0.037 and 0.038).

The overall complication rate was 10.1% and highest in the patients admitted at night (10.8%), followed by the weekend (10.5%), and then the day (9.4%), although this was not statistically significant (*P* = 0.9) (Table 1). Within the operative cohort, there was a 31% complication rate without any significant difference between the three admission times: (31.7%, 26.7% and 33.3% day/night/weekend, respectively; *P* = 0.82) (Table 1). The complications found in those patients who were not operated on were related to the admission diagnosis, of which the majority included Crohn’s flares, long term ileus, failure to thrive with nutritional deficiencies, and enteritis. The percentage of patients presenting with peritoneal signs was 0.9%. This was a rare presentation, however significant, and resulted in an operation in three of the four instances. The three patients went to the operating room immediately after presentation. Two patients were admitted during the day and two were admitted on a weekend. Three factors were associated with post-operative complications: history of coronary artery disease, history of chronic obstructive pulmonary disease and history of *diabetes mellitus* (*P* = 0.05, 0.02, and 0.04, respectively).

With regard to outcomes, the mortality rate for all patients was 1.7%: 0.4% for those managed non-operatively and 4.9% for those managed operatively (*P* = 0.005). There was no significant difference in mortality between the day/night/weekend groups when managed operatively (1.4%, 2.2%, & 1.7%, respectively; *P* = 0.35) (Table 1). There were no factors strongly associated with post-operative mortality.

## DISCUSSION

The routine management of patients admitted with partial small bowel obstruction, without overt peritoneal signs or sepsis, continues to be somewhat variable. Conservative non-operative management is generally accepted and recommended; however there is variability in deciding when medical management has failed and operative intervention is warranted. There are several studies, analysing management techniques and outcomes based on different variables [11–14]. Maung and colleagues recently published a practice management guideline for patients admitted with small bowel obstruction [13]. They stated that non-operative management is a safe option for stable patients who show no evidence of peritonitis, ischemia, or signs of sepsis. This practice management guideline also recommends considering either surgery or water-soluble contrast studies in those patients who have not resolved their obstruction by hospital day 3 to 5. Schraufnagel and colleagues recently analysed the effect of time to surgery in patients admitted with small bowel obstruction; they found an increase in post-operative mortality if there was a delay to surgery greater than four days [15].

There are likewise many other factors that have been associated with adverse outcomes in patients admitted with small bowel obstructions, including certain comorbidities, admitting service, and delay in admission or evaluation in the emergency room. Schraufnagel and colleagues found several comorbidities to be associated with increased complication and death [15]. In 2011, Huang *et al.* reported that delays in first evaluation by surgeons generally resulted in more patients undergoing surgical resection. Additionally, patients who experienced longer times to surgical consultation tended to arrive in the emergency room during shift changes [11]. Lastly, Oyasiju *et al.* reported an increase in morbidity and mortality in patients admitted to a non-surgical service [14]. While some of these studies do show an increase in morbidity and mortality in patients either admitted on a weekend or who experienced a delay to surgery, no study to date has analysed the impact of admission timing and outcomes in the management of small bowel obstruction.

The analysis of patient management during “off hour” times represents an important opportunity to improve patient outcomes. Certainly, if there are statistically significant findings, e.g. worse outcomes and inpatients admitted during the night and/or weekend, we should seek ways to improve and/or change patient management during these times. In multiple articles, we have reported examining the weekend effect of other disease processes: there has yet to be a unified consensus on whether time of admission affects a patient’s outcome. While one would expect patients to receive equally efficient care, regardless of their time of admission or presentation, multiple studies have shown this not to be the case, many citing worse outcomes in patients admitted on a weekend, particularly in scenarios in which timely intervention is probably directly related to mortality and morbidity [3–7, 16]. While other studies have not shown such a substantial difference, these appear to be in cases where urgent intervention was not required (medical admissions) or where set standards of care or algorithms (trauma systems) are in place [9, 10, 17]. In the cardiovascular literature, Nachal *et al.* recently reported a 20% increase in mortality among patients presenting on a weekend with acute pulmonary embolism, attributing this increase to delay in diagnosis and intervention [18]. Interestingly, other authors have looked into whether sicker patients tend to present at night or on a weekend, suggesting that the weekend effect could be attributed to more severe patient disease and not only on staff- or hospital shortcomings [19].

In our analysis of patients admitted from the emergency room with a diagnosis of small bowel obstruction, we did not find a difference in patient mortality or morbidity based on time of admission. Although patients experienced a delay in reaching surgery if admitted on a weekend, this did not correlate with increased morbidity or mortality. Although we did not analyse cost in this study, we would expect an increase in costs for night and weekend admissions, based on the significant delay before operative intervention in this group. Another interesting factor which may affect time to operation is changes in care staff. At our institution, the admitting staff generally care for the patients throughout their hospitalization; in an acute care surgery setting, changes in attending staff may result in delays in care or potential improvement in care: however that analysis was beyond the scope of our study.

This study complements others in respect of management and outcomes for partial small bowel obstruction patients. Patients tended to fair worse with certain comorbidities and, not suprisingly, have an increase in complications with operative intervention. Approximately 30% of our patients required an operation and, similar to other reports, the mortality rate was 1.7% for all patients admitted and 4.9% for those who underwent surgery [11, 14, 15]. Secondly, the only significant clinical finding among those patients requiring an operation were signs of peritonitis. But, interestingly, patients who required nasogastric tubes in the emergency room were more likely to require operation, suggesting that the surgeon believed they were probably in worse condition and thus predictive of surgical intervention. Also, those with elevated lactate and tachycardia were more likely to require a bowel resection, suggesting that these clinical findings may prove to be beneficial markers for deciding who should undergo surgical intervention.

Our study has a few limitations. This is a single-center, retrospective study and may reflect local and regional patterns of practice; the institution is also a residency training institution. Further, we sought to capture the timing of admission, but obviously there is a difference in the length of stay of patients in the emergency department. However, in order to keep the study balanced, we captured only the time of the actual first contact for general surgical consultation. The strengths of this study are that we were able to select variables *a priori* and also review the chart for emergency department specific demographics, in addition to standard variables.

## CONCLUSIONS

The analysis of certain time-sensitive disease processes—stroke, acute myocardial infarction, gastrointestinal hemorrhage and others—has demonstrated improvement related to the timing of intervention. In patients presenting to the emergency room with small bowel obstruction, despite a shorter time to operative intervention in those patients admitted during the day, our study revealed that time of admission (day *vs* night *vs* weekend) does not appear to correlate to patient outcome or mortality. This work supports previous studies with regard to outcomes in patients with small bowel obstruction, but tends to disagree with the assumption that patients admitted during a weekend and requiring urgent intervention tend to fair worse.

**Disclaimer:** the results and opinions expressed in this article are those of the authors and do not reflect the opinions or official policy of the United States Army or the Department of Defense.

**Funding:** The results and opinions expressed in this article are those of the authors, and do not reflect the opinions or official policy of the United States Army or the Department of Defense. The authors have no financial affiliations.

**Conflict of interest:** The authors report no proprietary or commercial interest in any product mentioned or concept discussed in this article.
